# Psychological Counseling during the COVID-19 Pandemic: Clinical Thoughts and Implications Arisen from an Experience in Italian Schools

**DOI:** 10.3390/ijerph19127255

**Published:** 2022-06-14

**Authors:** Yura Loscalzo

**Affiliations:** Department of Health Sciences, School of Psychology, University of Florence, 50135 Florence, Italy; yura.loscalzo@gmail.com

**Keywords:** counseling, Freud, Kalff, Jung, sandplay, sandplay therapy, school counseling, school psychologist

## Abstract

During the COVID-19 pandemic, I worked as a psychologist in two schools: a comprehensive school (an institution including three school levels: kindergarten, primary school, and secondary school of first grade) and a Provincial Center for the Education of Adults (CPIA). This paper provides some clinical considerations that arose from this personal experience, focusing on practical implications for school psychological counseling. Among the main points, I noticed that students were eager to disclose information about themselves in a professional space, were not afraid of being ridiculed by classmates for attending the service, and spontaneously used artistic media. Using English (a non-native language for both the Italian psychologist and the CPIA student) emerged as an added value for immigrant students who were not fluent in Italian. This allowed them to attend the psychology service and share their thoughts and feelings despite their difficulties with Italian. In conclusion, psychological counseling services should be implemented in all schools and across all school levels worldwide to favor psychological well-being and spread a culture prone to asking for psychological help. Moreover, using a non-native language might be helpful when working with international students. Finally, sandplay therapy (and art) might be an additional option to verbal counseling in school settings.

## 1. Introduction

A novel Coronavirus (COVID-19) characterized by high mortality and rapid transmission arose in China, Hubei, at the end of 2019 [1,2]. This virus was rapidly spread worldwide. In Italy, the first diagnosis was made in February 2020 in a little northern city of Lodi province [3]. Then Italy became one of the most affected European countries by COVID-19: the World Health Organization [4] registered 4,278,319 confirmed cases and 127,840 deaths from 3 January 2020 to 16 July 2021. Therefore, after a few gradual restriction measures, on 4 March 2020, the government adopted strict rules to contain the virus, including lockdown, the closure of the educational establishment, and the adoption of distance learning [5]. Unfortunately, the closure of the educational establishments lasted longer than expected since it also affected the new school year that started in September 2020. However, there were some variations across Italian cities concerning the closure’s length and timing in the school year 2020/2021. The closure of the educational establishment depended on both the educational level (i.e., kindergarten, primary school, secondary school of first grade, secondary school of second grade, and university) and the color associated with the level of risk (i.e., red: high, orange: medium, or yellow: low) of the region and even of the cities [6]. 

The impact of the educational establishment’s closure, with the consequent drastic reduction in social interactions, posed a great challenge for the well-being of young students. Pre-adolescence, which characterizes students in the secondary school of first grade, is a phase of the life cycle associated with many physical, psychological, and social changes due to the onset of puberty. At the end of puberty, adolescents reach skeletal maturity, fully develop primary and secondary sexual features, and fulfill reproductive capacity [7]. Pre-adolescence is a critical period for mental health due to the changes in this phase: psychological problems can arise and resolve shortly without intervention, but confirmed psychological disorders may also occur. For example, social anxiety disorder typically arises in adolescence [8]. However, scientific literature highlighted the importance of considering its onset at an earlier age, as timely detection has important preventive implications. Indeed, experiencing social anxiety, even in the absence of a formal diagnosis, is associated with negative consequences: feelings of loneliness, less pleasant time spent at school, greater use of negative stress management strategies (for example, worry and guilt), and lower academic skills [9]. In the Italian context, the need to prevent its onset is of foremost importance: recent data showed that Italian adolescents have high levels of social anxiety [10]. 

Therefore, it is essential to prevent the onset (or chronicization) of psychological problems that could arise during pre-adolescence. Considering the current historical moment characterized by a global health pandemic, it is even more critical. The COVID-19 outbreak forced pre-adolescents (and children and adolescents) to remain indoors for a long time and interact with friends and classmates only through social networks. From puberty, there is an increase in relationships between peers (both on a friend and sentimental level). A good relationship between peers institutes an index of psychological well-being and is a protective factor for psychosocial issues [11]. Reducing the opportunities for social contact both in the classroom and outside the school can limit social relationships; therefore, it might lead to negative consequences for pre-adolescent health.

For children—that is, primary school and kindergarten students—the consequences of the pandemic, apart from problems in school learning (especially in the case of those with certified neurodevelopmental disorders), may have also led to psychological issues. Children can experience anxious and depressive states such as pre-adolescents and adolescents. Though, compared to them, they may have greater difficulty recognizing and verbalizing their emotional states; hence, they may express their disturbing feelings mainly on a somatic level. Consequently, identifying the underlying psychological disorder is difficult [12]. Furthermore, the educational establishment’s closure reduced the hours required for lessons and homework and, simultaneously, increased the time available for video games and video calls to friends. This could have caused an alteration in the daily routine (i.e., getting up early, going to school, doing homework, and then going to bed early), with a consequent alteration in the quantity and quality of sleep. In children, this can have highly damaging effects such as a reduction in impulse control and attention capacity, the onset of hyperactivity [13], and an adverse effect on immune functions [14]. 

In sum, psychological counseling for children, pre-adolescents, and adolescents—possibly located at schools and without cost to families—is fundamental to facilitating the maintenance (or recovery) of their psychological well-being, especially during a health crisis such as the one we are still living. Though, about school psychological counseling, there are some differences among different countries, including the legislative framework, the position requirement, the functions of the school counselor, and the number of students per counselor [15]. Among the countries that Popov and Spasenovic [15] analyzed, this ratio averagely varies between 250 and 500, with the best standard in Croatia: two counselors for schools with about 180 students and four counselors for institutions with more than 500 students. In the USA, the average is 250 students for a counselor. There are countries, such as Bulgaria and Russia, whose standard is instead 500 students per counselor [15]. 

In Italy, according to the public calls spread in the last years, schools usually enroll one school counselor for all the students of the institution, even in the case of comprehensive schools, which include three education levels (i.e., kindergarten, primary school, and secondary school of first grade). In line with this, Italy does not figure out among the 39 countries (out of 82) in which school-based counseling is mandatory. Among the countries that have this service mandatory are Austria, Japan, Turkey, Ghana, France, and 32 (of 50) states in the USA [16]. Concerning Western Europe, Harris [16] underlines that counseling is not well developed as a profession in Italy. 

Therefore, the Memorandum of Agreement signed by the Italian government in 2020 represents an important step towards developing and spreading school counseling in Italy. More specifically, on 16 October 2020, the Italian Ministry of Education signed a Memorandum of Agreement with the National Council of the Order of Psychologists. This Memorandum aimed to provide psychological support to school staff, students, and families of all Italian schools (from kindergarten to secondary school of second grade), during the school year 2020/2021. It aimed to respond to the traumas and discomforts deriving from the COVID-19 emergency and, at the same time, prevent the onset of psycho-physical distress. Regarding funds, the government provided EUR1600 for 40 h of psychological service. The Memorandum established a compensation of EUR40 per hour. Next, if the schools proved to have used this first tranche of money, the government gave EUR3200 for an additional 80 h. Hence, this Memorandum allowed schools to implement 120 h of psychological service in 2020/2021 [17]. Thanks to this Memorandum, 69.22% of Italian schools implemented a psychological counseling service in their institution (that is, 5662 up to 8183 schools); for most of them (3178), this was the first time they provided a counseling service. The other schools continued or potentiated a previous psychological service [18]. 

I have been personally involved in this agreement, as I carried out the psychological counseling service in two schools: a comprehensive school (that is, an institution including three school levels: kindergarten, primary school, and secondary school of first grade) and a Provincial Center for the Education of Adults (or CPIA), which includes courses addressed to foreign people who need to learn the Italian language. Hence, through this paper, I aim to provide some clinical considerations that arose from this personal experience, focusing on practical implications for school psychological counseling.

## 2. Clinical Thoughts 

### 2.1. Straight to the Point!

Freud [19], in his introduction to the clinical case of the “wolf-man,” presented the topic of the importance of setting a deadline for the analysis, at least in some cases. This patient was very resistant to the treatment. After a long time spent without progress, as soon as he reached some amelioration, he stopped his commitment to the therapy, which prevented moving further with his well-being. Hence, once the transfer was established, Freud decided to declare to the patient his resolute decision: they would have ended the treatment once they reached a specific date, regardless of the progress made. Next, a sudden movement happened in the therapy: his resistance to the psychoanalysis and his attachment to the symptoms fell; in a short time, he gave all the information needed to solve his inhibition and dissolve his symptoms. 

While reflecting on the work I made with pre-adolescents at school, this introduction of Freud [19] popped up in my mind. In my experience, the psychological service provided short sessions of 25 min. During the first meeting, despite parents already knowing the counseling service’s timing, I informed the student that our time together would be limited in length of the session (i.e., 25 min) and counseling service (i.e., number of sessions). I could not provide a pre-established number of sessions. I had to be available to receive new requests during my stay at the school. Moreover, once I reached nearly half of the hours initially committed, I was informed that the contract would probably be integrated with more hours to meet the number of requests received, thanks to the additional funds provided by the government. Hence, I informed the students that we would meet at least three times but that, based on the requests from other students and the possibility of extending my working hours, there was the possibility of increasing the number of sessions. Nevertheless, I clarified that I would have informed them in advance about the last session: when reserving the last spot available for them, I would have specified that the next meeting would also have been our leaving time.

In summary, considering some school-related logistic limitations for my counseling service, I found myself posing some restrictions to the students asking for my psychological support. Interestingly, students were willing to participate despite these restraints, and I felt that it happened just as Freud [19] presented in his wolf-man case. There might have been other individual and environmental variables—that I did not measure quantitively—influencing the students’ openness. However, during my work with them, I felt a sense of urgency in most students: since their first meeting, it was as if they were throwing up an intertwined skein of events, memories, dreams, doubts, and hopes. It was as if they had long waited for a space in which they could say what was dear to them. Now, grasping the limited space allowed them, they did not become lost in pleasantries and went straight to the point. What struck me was the large and significant amount of information confidently offered to me from the first session together, and especially the clarity with which youths disclosed so many topics. They allowed me to follow them in the intertwining of their threads since they presented the information in an orderly way, one at a time. At the end of the session, it seemed as if they had taken off a long-held weight.

#### Clinical Implications

Logistic-imposed limitations, which are present when the psychologist does not work in the private office, might have a positive effect: they might stimulate the student to disclose inner thoughts and emotions quicker. If psychologists accept the restrictions of the counseling school service, they might be an essential resource for students. What seemed important to me with students was not “doing something” or reaching an aim (such as removing a panic attack or a crisis of anger) but instead staying emotionally with them during the time we had the chance to be together. There have been moments in which we did something together (breathing exercises, usually), but what I felt they needed the most was someone open to listening to them without judgment. As a second point related to this, it sharply emerged that pre-adolescents need to be listened to in a professional space, in line with recent data highlighting an increase in requests for psychological support among Tuscan adolescents during the COVID-19 pandemic [20]. Although they have parents, friends, and social media, they need to have a person in front of them who is open to listening to them and making them feel that they are fine, whatever they think, dream or fear. They certainly need to argue and have support from parents and friends about their ideas and feelings, but they also need a place to rest for a while and feel free to be what they want without the fear of being judged. 

### 2.2. Going to the Psychologist Is for “Crazy People”… or Maybe Not?

In private practice, it is usual to receive the first contact from parents, with the first phase of counseling usually dealing with motivating the minor to be there. Though, in my recent experience as a school psychologist, I received a few e-mails from the student asking for an appointment. In these cases, I informed them that it was necessary to have the written consent of their parents to make an appointment. However, I believe that this voluntary act made by some of these pre-adolescents indicates that they were firmly willing to have a space to disclose themselves. This should not be overlooked since it might suggest a strong need to be empathically listened to, even when they fear that parents might disagree with their choice. 

Interestingly—even if I want to underline that all the parents I met were positive (and proud) about their child’s request—in some cases, I perceived the surprise from the parents about the willingness of their child to come to the psychological service. The students attended the service during school hours: their classmates would have understood that they were going to the psychological service; hence, they feared that this would have led to them being ridiculed by the class. However, the students, *at least those who asked for the service*, did not seem to be worried about the risk of stigmatization that could have been associated with asking for psychological support. Moreover, it is interesting to note that once a student came to the service, other scholars from the same class usually asked for an appointment. I speculate that spreading the psychological service at school could lead more and more children and youths to be more prone to see psychologists as a resource and not as doctors to be seen “in secret” (to avoid stigmatization from peers) and just in case of severe symptoms (such as a panic attack, acute depression or eating disorders) that could better justify the request for help. Consequently, psychologists could be looked for in case of mild symptoms or just for talking with someone who is not a relative or friend, with positive implications for the well-being of the student in the short term and the prevention of the arising (or chronicization) of clinical disorders in the long term.

I did not collect quantitative or qualitative data about the students’ fear of stigmatization. I based my speculation on students who asked for the service and knew they would have attended it during school hours, leaving their class in front of classmates and teachers. It is also possible to hypothesize that the pandemic might have played a role in destigmatizing mental illness and requesting psychological help. A recent survey conducted by order of Psychologists of Tuscany concerning the period between March 2020 and February 2022—involving 1099 Tuscan psychologists—showed that 67.2% of Florentine psychologists reported increased requests compared to the period prior to COVID. There have been increased requests for 81% of Tuscan psychologists about adolescents specifically. These adolescents reported as primary problems anxiety and depressive symptoms, relationship problems, self-harm, and eating disorders [20]. In line with this, students from all three years of secondary school attended the service (and I did not detect age-related differences in the willingness to return to the service). However, most of them were from the last class (i.e., between 13 and 14 years), corresponding to the school year nearest to the adolescent age.

Remarkably, even when the students came to the service due to the parents’ or teachers’ will (in this case, there was also parents’ agreement) and not by their own will, I found them interested in the service. After a few minutes of ice breaking, they seemed happy to have the occasion. In addition, when I asked them if they would have liked to come back at the end of the first meeting, they always replied positively. This indicates that it might be helpful to have an intercession from parents and teachers. They might support students to access a space they could appreciate, even if they could be unwilling to ask personally for many different reasons (e.g., fear of the unknown, fear of stigmatization, fear of questions by the parents, teachers, or peers). Related to this point, I think it is noteworthy to specify that I also received a few children from primary school, thanks to the parents’ motivation to take them to the service (for children, the length of the session was longer: 40 min). When asked if they wanted to come back after the first session, even children replied positively (and went straight to the point, such as the students from secondary school). Finally, in addition to students presenting with anxiety, depression, and anger issues, I also received students who asked to attend the service—or who decided to keep coming even if they did not ask personally—that used their time to talk about their friends, their loves, their dreams, their hobbies. This further highlights the critical need for pre-adolescents to be listened to by someone who is not a parent, a teacher, or a friend but an unrelated person with empathic competencies and the duty of professional secret. 

#### Clinical Implications

First, these considerations suggest the importance of providing as many occasions as possible for pre-adolescents (and children and adolescents) to talk about themselves in an atmosphere of professional empathy. Pre-adolescence is a critical stage of life associated with many physical, psychological, and social changes [7,21,22]. Hence, also in the absence of a health pandemic, it is vital to provide students with a space in their schools to be listened to and supported in their growth by a psychologist. Second, the possibility of having their first psychological experience at school might help students ask more easily for professional help in the future if they need treatment, even for mild symptoms. In line with this, spreading the psychological counseling service at school (and consequently a positive attitude towards the psychologist) might help reduce the fear of the stigmatization that, unfortunately, is still present to some extent, and that might be an obstacle in receiving timely help. Finally, as pointed out by the willingness to have other meetings even when the students came after being prompted by the parents or teachers, the critical role that the adults surrounding students arises in that they might help them obtain the support they need. Sometimes, students do not know about the possibility of having someone at school with whom they can have a professional talk; sometimes, they are scared about asking for this service; sometimes, youths are afraid about their classmates’ reactions. Hence, parents and teachers might help them make the first step toward the service. Furthermore, whenever they face “difficult students”, I strongly suggest teachers ask them to use the psychological service: usually, angry students are youths who feel they are not listened to and struggle for attention. Thanks to the teachers’ willingness to help them and the psychologist’s empathy, they can obtain this strive for attention. 

### 2.3. Not Only Italian Words, but also Drawing, Songs, and Non-Native English Language

Although we had less than half an hour in each session, some pre-adolescents (especially third-year students) used this time to draw or bring me some drawings to contemplate or discuss together. Sometimes, I felt they used drawings to establish a relationship with me, while at other times, I felt they used illustrations to better conveys their feelings any time words were not enough for them. Another artistic medium used is music, more specifically English songs. Some of these pre-adolescents had a good knowledge of English, and they used it as a resource to express their deep feelings through the words of their favorite singers. 

Regarding English, I would like to add a reflection regarding my experience at CPIA. In this institution, there are Italian adults (and a few adolescents); however, there are especially immigrant adults needing to learn the Italian language who sometimes can speak English thanks to their education in their home country. This school allocated me more working hours (i.e., all the hours available through the Memorandum of Agreement), and the number of students was lower than in the other school; hence, each session lasted 45 min. In this context, I welcomed the request for psychological counseling from adults who could not speak Italian thanks to the opportunity to use the English language with them. It might be questioned that using a non-native language learned at school (for both the person asking for help and the psychologist) might be challenging and lead to a more rational conversation. Based on my personal experience, I can affirm instead that the psychologist can establish a profound connection and an emotional conversation also in this case. Empathy is mainly conveyed through body language, and words are just a means to establish a conversation and relationship. For example, feeling free to cry in front of someone who professionally cares about you and does not morally judge you for your acts, feelings, and thoughts might alleviate the pain of a difficult life lived in solitude (even if amid other people). The healing process might occur even if the psychologist does not understand every single word or the person cannot express his/her feelings in the native language. 

#### Clinical Implications

Given the use of drawing and music naturally applied by pre-adolescents, I suggest incorporating these artistic media into school counseling practice. More specifically, when the students present the psychologist with a song or a drawing to express their feelings, it might be valuable to ask them to bring a song or another drawing they would like to share at the next meeting. This could help deepen the relationships and have the students become more involved in the psychological reflection about themselves between the sessions, even if these sessions are brief or at a very long interval of time. Second, thinking about the CPIA experience specifically, I encourage psychologists who are confident with their English—or, more generally, with a non-native language (even if it is the so-called “learned at mandatory school language”)—to avoid the language being a barrier in their work with international students and try to use it to establish a connection with the person. If both the student and the psychologist are involved in the conversation using a non-native language, it might be reduced the client’s feeling of being judged for his/her poor competence in the psychologist’s language. Furthermore, the collaborative efforts to understand each other’s English (or another non-native language, in the case of psychologists whose mother tongue is English) might help build the relationship. 

## 3. Discussion

Based on my personal experiences and the clinical thoughts that arose from them, I suggest that Italian schools, which implemented psychological counseling services thanks to government funds, will continue this service in the following years (using their funds if needed). My experience highlighted that children, pre-adolescents, and immigrant adults strongly need spaces devoted to listening to them in an atmosphere of professional empathy. When they can use this service for free at school, they ask for appointments or keep coming even if they are prompted, at first, by parents or teachers. Hence, I invite countries that do not utilize psychological service in all their educational establishments, such as Italy, to consider its critical need for students and implement it. For these countries, it would be necessary for their government to establish that school-based counseling should be mandatory. Regarding CPIA (i.e., a school for the education of adults, including immigrants needing an Italian language certification), the psychological service might be of great added value since adult immigrant people usually have economic issues that prevent them from asking for professional psychological help. Furthermore, in some cultures especially, it might be hard to disclose the attendance of this service to the family, even if it is for free. For example, a recent study showed that Pakistani have some barriers to seeking psychological help, including social defame, personal shame, and prohibition by the family [23]. Picco et al. [24] highlighted that Malay ethnicity is associated with lower openness to seeking professional help; in addition, Malay and Indian ethnicity is associated with a lower value assigned to seeking professional help. Regarding the USA, Cheng et al. [25] found that Asian Americans were less favorable toward seeking psychological help than other ethnicities. Therefore, having the possibility to have psychological counseling at school is a great opportunity for CPIA students: they do not have to pay for this service, and, most importantly, they might attend it during school hours without having to disclose this (if they do not want to) to their partner or other family members. 

Moreover, spreading the psychological counseling service at school is essential to favor a culture prone to consider psychologists as a resource for the well-being instead of as a professional to be looking only for severe symptoms due to the shame of asking for help (especially if the person does not have frank signs of anxiety, depression, or other clinical symptoms). The students who come to the psychological service can demonstrate to their classmates, teachers, and parents that asking for help is fine. They constitute a role model for those worried about the stigmatization that could arise by attending the psychological service. Therefore, implementing the psychological counseling service in all the schools and at all schools levels (including colleges and CPIAs) might constitute a crucial step towards the prevention of psychopathology: if children, pre-adolescents, and adolescents feel free to ask for psychological help (at school or, if needed, outside the school for treatment) as soon as they feel the need, it will be possible to prevent the onset of disorders or their chronicization. 

A practical implication also concerns using English or other non-native languages known by the psychologist (especially for psychologists whose mother tongue is English but who work in schools or colleges with international students) during psychological counseling. It constitutes an extraordinary opportunity for international students. If the psychologist can use a non-native language that the student also knows, this might help those who do not know well enough the psychologist’s mother tongue to access the psychological counseling service. They might avoid using the language they are learning and use instead another language already studied by them during their education in their country of origin. 

Finally, based on my inclination to the sandplay therapy technique, I suggest implementing it in school psychological counseling services (if the psychologist is adequately trained in its use) through an office dedicated specifically to the psychologist, in which he/she might put the sandtray and the shelves with miniatures. This might be an added value for primary school children, for whom play techniques might be more easily applied than verbal counseling. Moreover, it might be advantageous for pre-adolescents and adolescents (who used expressive and artistic techniques spontaneously) and immigrant people (including college students and adults, who might have difficulties with the psychologist’s mother tongue). Even if I did not apply sandplay therapy during my experience in these schools, a vast scientific literature supports its efficacy, including its use through group sessions [26,27,28]. Regarding the cost of implementing sandplay therapy in school counseling services, there should be a room that is not used for other school activities. Moreover, accordingly to a U.S. website for play therapy supplies, a wooden sandtray with a stand and sand costs around USD500, and a miniature shell around USD200. Finally, with about USD300, it is possible to have a basic set of miniatures covering the following categories: people; structures (e.g., town buildings, a school, houses); fantasy; battle and warfare; death, grief, and faith; transportation; landscape and natural elements; animals; containers; leisure and household. Hence, the total cost is around USD1000 (plus shipping cost), which might be slightly higher or lower depending on the number of miniatures selected. However, once bought all the supplies needed, there are no further expenses in the following years, except, eventually, new sand to be put in the tray or a few new miniatures which stand out as essential to be included based on the work in the school.

Moreover, I suggest using artistic media as an additional resource (such as drawing and songs) to talk therapy. As Newcomb and Centeno [29] highlighted, counselors frequently use art in counseling, and there is increasing recognition that it is an essential venue for healing. There are two different art uses in counseling: as an expressive tool adjunct to the talk therapy process and as a therapy process with its own goals [29]. Based on my experience—which highlighted that some students used them naturally as an adjunct to their talk, even in the lack of materials offered to them—I suggest having in the office the basics for drawing: sheets of paper, a pencil, a rubber, a pencil sharpener, and colors (pencils and pens). This drawing set is economical since it might be arranged for less than USD100 and used for the entire school year. During the first meeting, the psychologist might introduce the students to the possibility of using their time during the service for talking, drawing, or doing both simultaneously. Furthermore, if the students mention a song that might represent their feelings or thoughts, the psychologist might suggest they print the song’s text and bring it to the next session to work on it. Apart from talking about the feelings and thoughts expressed by the song, it is also possible to ask them to do a picture about that song since this might help deepen their insight about feelings and thoughts. Using pictures and songs is feasible even in the absence of an office specifically devoted to the psychological service. It might help build the relationship and favor the students’ reflection on their feelings, thoughts, troubles, and solutions during the time between the sessions with the psychologist.

In conclusion, I recommend that all schools worldwide (and all school levels) have a psychological counseling service. Moreover, it would be essential to have the psychological service available for the entire school year (for Italy, it would mean from September to June) and for as many hours as possible. In this vein, it would be important that governments establish the duty of a school counseling service in all the educational establishments and hence provide public schools with funds for this service. The Italian Memorandum of Agreement signed in 2020 provided all schools with a maximum of EUR4800 for 120 h of psychological service for the school year 2020/2021. Hence, on average, the schools could offer four hours per week of psychological service, mainly devoted to individual counseling. It would be advisable to have funds for at least 240 h per year, so to allow having more space for individual counseling but also for other psychological activities, such as class observation (frequently asked by teachers to have educational guidance), complex cases discussion, and class activities to improve students’ soft skills. However, this would require receiving at least EUR9600 per year from the government.

Also, it would be advisable to organize a psychological service devoted office to settle a sandtray and miniatures on shelves (or, at least, art and play therapy supplies) to be used as an additional option to verbal counseling. Finally, it would be important to spread in all countries worldwide the institutionally recognized figure of the “school psychologist,” namely a licensed psychologist with proper expertise in school counseling and who is a school employee (like teachers) working at school for all school hours and with continuity from a school year to another. Considering Italy, to the best of the author’s knowledge, currently, school psychological services are *usually* committed through annual public competitions and with the following order of selection: (i) teacher working in the school but who also has a license as a psychologist; (ii) teacher working in another school but who also has a license as a psychologist; (iii) an external professional, namely a psychologist who does not work in schools as a teacher. The psychologist might have different competencies and experiences in educational/school psychology (based on the evaluation criteria selected by each school individually), and the number of hours allocated varies across schools (especially outside of the Memorandum of Agreement). I believe, instead, that it would be essential to guarantee continuity in the school psychological service by having, if possible, the same psychologist in different school years for these reasons: (i) the psychologist might acquire knowledge concerning the organization of the school, including the relationships among the employees, with critical effect on his/her work in the subsequent years; (ii) the students and the teachers, once built the relationship with the psychologist in the first year, might continue to receive support from the same person instead of beginning a new path with another one. Moreover, if psychologists were included among the employees of the schools, they could be present for all school hours, not just a few hours per week (this also applies if the psychologist is a schoolteacher also involved in the psychological service since he/she works in the counseling service outside his/her lessons’ hours). This full-time presence would guarantee a better psychological service: the psychologist could be available, on-demand, also for prompt interventions or for group interventions in class (which have been, in my experience, frequently asked by teachers). Furthermore, it would be useful to have psychologists in the same school of a different gender. This would allow students to choose the therapist according to their gender, which might be especially important for adults from cultures that are sensitive to gender-related differences. However, at least in Italy, gender might not be used among selective criteria since they must be based instead on competencies.

About the limitations and strengths of this paper, the recommendations provided are based on clinical thoughts that arose from a personal experience; no quantitative or qualitative data support them, nor have I offered clinical data concerning specific students. However, as a commentary (not research) paper, it has the main merit of having proposed a critical reflection concerning the usefulness of the counseling service at schools during the difficult time posed by the COVID-19 pandemic, with important implications for the practice of school counseling also in future (non-pandemic) times.

## 4. Conclusions

During the COVID-19 pandemic, I conducted the psychological counseling service in two schools: a comprehensive school (including kindergarten, primary school, and secondary school of first grade) and a Provincial Center for the Education of Adults (CPIA). This paper provides some clinical thoughts and implications that arose from this personal experience, focusing on practical implications for school psychological counseling. Among the main points, I found that children, pre-adolescents, and immigrant adults strongly need spaces devoted to listening to them in an atmosphere of professional empathy. Furthermore, the presence of a school counseling service seems to support the spread of a culture prone to consider psychologists as a resource for well-being. Moreover, it emerged the worth of using English, or other non-native languages, during psychological counseling. It constitutes an opportunity for immigrant and international students looking for psychological support who are unfamiliar with the psychologist’s mother tongue. Finally, I suggest implementing the sandplay therapy technique, or at least artistic media, as an additional resource in school-based counseling.

## Data Availability

Not applicable.
